# Comparison of oxygen reserve index according to the remimazolam or dexmedetomidine for intraoperative sedation under regional anesthesia—A single-blind randomized controlled trial

**DOI:** 10.3389/fmed.2023.1288243

**Published:** 2023-11-15

**Authors:** Sangho Lee, MinSung Kim, Hee Yong Kang, Jeong-Hyun Choi, Mi Kyeong Kim, Ann Hee You

**Affiliations:** Department of Anesthesiology and Pain Medicine, Kyung Hee University College of Medicine, Kyung Hee University Hospital, Seoul, Republic of Korea

**Keywords:** dexmedetomidine, heart rate, oxygen reserve index, peripheral oxygen saturation, randomized controlled trial, regional anesthesia, remimazolam, sedation

## Abstract

**Introduction:**

We aimed to evaluate the difference in intraoperative oxygen reserve index (ORi) between the sedatives remimazolam (RMMZ) and dexmedetomidine (DEX).

**Methods:**

Seventy-eight adult patients scheduled for sedation under regional anesthesia were randomly assigned to either the DEX (*n* = 39) or RMMZ (*n* = 39) group. The primary outcome was the difference in perioperative ORi between the groups. The secondary outcomes included respiratory depression, hypo- or hypertension, heart rate (HR), blood pressure, respiratory rate and postoperative outcomes. Additionally, the number of patients who experienced a decrease in intraoperative ORi to < 50% and the associated factors were analyzed.

**Results:**

The ORi was significantly higher in the RMMZ group at 15 min after sedation maintenance. There were no significant differences in respiratory depression between the two groups. The intraoperative HR was significantly higher in the RMMZ group after the induction of sedation, 15 min after sedation maintenance, and at the end of surgery. No other results were significantly different between the two groups. The incidence of a decrease in intraoperative ORi to < 50% was significantly higher in the DEX group. Factors associated with a decrease in the intraoperative ORi to < 50% were diabetes mellitus, low baseline peripheral oxygen saturation (SpO_2_), and DEX use. In the receiver operating characteristic curve analysis for a decrease in the intraoperative ORi to < 50%, the cutoff baseline SpO_2_ was 97%.

**Conclusion:**

RMMZ is recommended as a sedative for patients with a low baseline SpO_2_ and intraoperative bradycardia. Further studies should be conducted to establish the criteria for a significant ORi reduction.

## 1. Introduction

Regional anesthesia is preferred over general anesthesia because of its various advantages such as less cognitive dysfunction, faster recovery, and reduced respiratory complications (1). When surgery is performed under regional anesthesia, a sedative is administered to reduce patient anxiety and discomfort (2). Traditionally, small doses of midazolam and propofol are administered for sedation (3). However, since these drugs may induce severe respiratory depression or drops in blood pressure (BP), careful patient monitoring is required (4, 5).

Dexmedetomidine (DEX) is a relatively safe drug with weak respiratory depression effects and is currently widely used in clinical practice (6, 7). As an alpha-2 agonist, it induces a sleep pattern similar to physiological sleep (8). However, loading-dose infusion may induce hemodynamic deterioration or delay emergence from sedation because of its long lasting action (9, 10).

Remimazolam (RMMZ), is a recently developed very short-acting, intravenous infusion-based benzodiazepine (11, 12). Similar to midazolam, RMMZ acts on gamma-aminobutyric acid A receptors to induce sedation or anesthesia (13). Additionally, the duration of action is short and predictable owing to its fast on- and offset. Similar to other benzodiazepines, sedation can be reversed using flumazenil (14, 15), and research on its hemodynamic stability is being actively reported (16). However, high doses of RMMZ also cause respiratory depression (17).

Respiratory monitoring is essential during sedation because of the possibility of respiratory depression caused by sedative agents. The risk factors for respiratory depression are advanced age, female sex, obstructive sleep apnea, chronic obstructive pulmonary disease, cardiac disease, diabetes mellitus (DM), hypertension, neurologic disease, renal disease, and obesity (18, 19). Generally, peripheral oxygen saturation (SpO_2_) and end-tidal carbon dioxide are monitored to detect respiratory depression (20). However, the oxygen reserve index (ORi), a recently developed non-invasive continuous parameter, can provide a better oxygenation profile since it reflects an increase in blood oxygen partial pressure within a range that does not reflect SpO_2_ (21, 22). Rather, ORi represents a blood oxygen partial pressure range of 100–200 mmHg assigned a numerical value of 0–1 (23). Previous studies have used ORi to adjust the perioperative fraction of oxygen supply (24, 25). Research related to the early warning of desaturation using ORi has also been actively conducted (21–23, 26, 27). ORi provides an early warning of the occurrence of desaturation, approximately 30–90 s earlier than SpO_2_. When monitoring patients in the operating room, the ORi can be used to predict and prepare for desaturation in advance. If these early warning features of ORi are used for sedation, anesthesia can be administered safely by maintaining the patient oxygenation and preparing for desaturation (28, 29).

However, studies comparing the ORi between DEX and RMMZ during sedation have not yet been conducted. Therefore, the authors of this study hypothesized that there would be a difference in intraoperative ORi according to the use of DEX or RMMZ during sedation and attempted to evaluate this difference using a randomized controlled trial. In addition, we assessed whether postoperative outcomes differed depending on the drug used.

## 2. Materials and methods

### 2.1. Study design and ethical approval

This study was designed as a single-blind randomized controlled trial to compare the ORi between two groups during sedation with DEX or RMMZ. Ethical approval was obtained from the Institutional Review Board of Kyung Hee University Hospital (KHUH 2023-02-036) on March 17, 2023. The study complied with the principles of the Declaration of Helsinki and followed the Consolidated Standards of Reporting Trials checklist. Before enrollment, the study was registered with the Clinical Research Information Service (No.: KCT0008339; registration date: April 07, 2023; principal investigator: Ann Hee You). The study protocols are available from the Clinical Research Information Service. Written informed consent was obtained from all participants.

### 2.2. Participants

This study included adult patients aged between 18 and 100 years who were scheduled for elective surgery under regional anesthesia and sedation at a single tertiary hospital. The exclusion criteria were pregnancy, allergy to the study drugs, preoperative oxygen supply, inability to communicate, non-supine surgical posture, severe obesity with a body mass index > 35 kg/m^2^, and American Society of Anesthesiologists physical status class ≥ IV. Participant recruitment began in April 2023 and ended in July 2023.

### 2.3. Randomization and blindness

Participants were randomized into the DEX or RMMZ groups on the morning of surgery using sealed envelopes. A random allocation sequence was generated with 1:1 allocation and random block size using Excel 2019 (Microsoft). Owing to the difference in the drug administration method, we were unable to fully double-blind this study; rather, the study was single-blinded since only patients were unaware of group assignment. However, postoperative outcomes were evaluated by a single researcher (MK) who was blinded to the group assignment.

### 2.4. Study protocol

After patients entered the operating room, a Masimo Radical 7 pulse oximeter probe (Masimo Radical 7; Masimo Corp., Irvine, CA, USA) was attached to the patient’s finger to measure the ORi. Heart rate (HR) and rhythm were monitored using a 3-lead electrocardiogram, and BP was measured every 5 min non-invasively using an arm cuff. Brachial plexus block and spinal anesthesia were performed for upper and lower extremity surgeries, respectively. After regional anesthesia, oxygen was supplied at 6 L/min via a non-rebreathing facial mask during the entire sedation. Before study drug administration, preoxygenation was performed to measure the ORi plateau value as a baseline. The modified observer’s assessment of alertness/sedation (MOAA/S) scale was used to evaluate the depth of sedation (30, 31). During the induction and maintenance of sedation, the sedative dose was adjusted to a target MOAA/S scale 3. In the RMMZ group, 2.5 mg was administered intravenously over 1 min to induce sedation; when a MOAA/S scale score of 3 was not reached, an additional 2.5 mg was administered. During maintenance of sedation, RMMZ was continuously administered intravenously at a rate of 0.1–1 mg/kg/h. In the DEX group, 1 μg/kg was intravenously administered for 10 min to induce sedation, and continuous intravenous infusion was administered within the range of 0.2–0.7 μg/kg/h to maintain sedation. The TERUFUSION^®^ INFUSION PUMP TE-171 (Terumo^®^, Tokyo, Japan) infusion device was used in both groups. Sedatives were discontinued during the surgical wound dressing stage.

### 2.5. Outcomes

The primary outcomes were defined as the difference in ORi between the two groups during sedation. ORi was measured as the baseline value at the plateau level in the preoxygenation stage, after the induction of sedation, 15 min after sedation maintenance, and at the end of surgery. Most ORi values in room air were 0, and the ORi plateau level in the preoxygenation stage before the administration of sedative drugs was set as the baseline.

Cases of respiratory depression during sedation were evaluated as the secondary outcomes. Respiratory depression was defined as a decrease in SpO_2_ due to the cessation of spontaneous breathing. HR, BP, and respiratory rate (RR) were measured at the aforementioned time points and in the post-anesthesia care unit (PACU). A change of > 20% compared with the baseline systolic BP was defined as intraoperative hypo- or hypertension, and the incidence of both was evaluated. The RR was measured through end-tidal carbon dioxide monitoring. Regarding postoperative outcomes, the PACU length of stay (LOS), postoperative nausea and vomiting (PONV), delirium, acute kidney injury (AKI) until postoperative day 2, and postoperative hospital LOS were evaluated based on medical records. AKI was defined as an alteration of postoperative serum creatinine ≥ 0.3 mg/dL compared to the preoperative level, according to the kidney disease: improving global outcomes criteria (32). Additionally, the number of patients who experienced a decrease in intraoperative ORi to < 50% compared to the plateau level during the preoxygenation stage and associated factors were evaluated.

### 2.6. Sample size calculation

Chen et al. (33) reported a minimum value of SpO_2_ of 88.22 ± 2.16 and 89.90 ± 2.03 when RMMZ and DEX were administered as sedatives, respectively, during bronchoscopy. Based on these results, 35 patients were included in each group as a G-power analysis (*t*-tests, means: difference between two independent means [two groups], *a priori*: compute required sample size–given α, power, and effect size, two tails, effect size d 0.802, α err 0.1, power 0.95, allocation ratio N2/N1 1). Considering a predicted dropout rate of 10%, the total target number of patients in the current study was 78, with 39 patients in each group.

### 2.7. Statistical analysis

Data are presented as medians [interquartile ranges] or numbers (%), as appropriate. The normality of continuous variables was evaluated using the Shapiro–Wilk test. Independent variable *t*-tests or Wilcoxon rank–sum tests were used to analyze continuous variables. The chi square or Fisher’s exact test was used for categorical variables. Univariate logistic regression analysis was used to identify the factors causing a decrease in the intraoperative ORi to < 50% compared with the plateau value during preoxygenation; all variables with *p* < 0.2 and previously described clinically important factors were included in the multivariate analysis. Receiver operating characteristic (ROC) curve analysis was performed to identify factors predicting a decrease in the intraoperative ORi to < 50%, and the cutoff value was obtained using the maximum Youden index (sensitivity + specificity–100). For all data, statistical significance was set at *p* < 0.05. Statistical analyses were performed using the commercial statistical software SPSS (version 22.0; SPSS Inc., Chicago, IL, USA).

## 3. Results

### 3.1. Study population and demographic data

**FIGURE 1:**
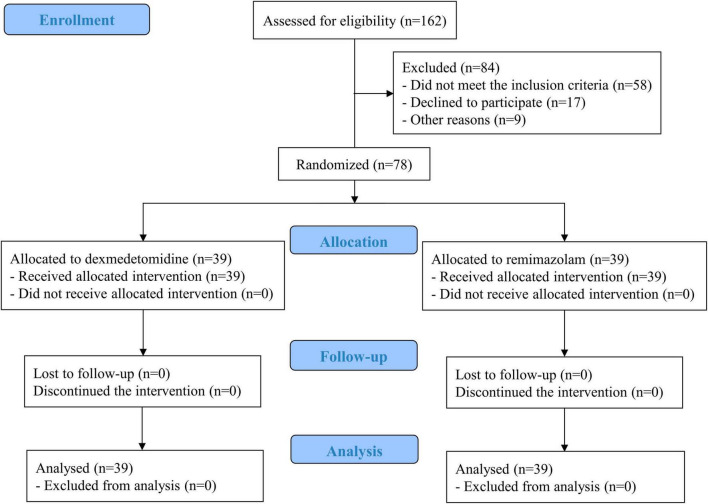
Flow chart of patient selection.

Of 162 patients, 84 were excluded, and 78 were randomly assigned to either group while ensuring both groups were the same size. The analysis was performed on 39 patients in each group without any loss to follow-up (Figure 1). There were no significant differences between the two groups in demographic data, medical history, preoperative laboratory test results, surgical site, amount of fluid administered during surgery, operative time, or intraoperative sedation level based on the MOAA/S scale (Table 1; Supplementary Tables 1, 2). No serious complications related to anesthesia or surgery occurred during the study period.

**TABLE 1:** Demographic and intraoperative data of the study cohort.

	DEX (*n* = 39)	RMMZ (*n* = 39)	*p*-value
Age (year)	58 [47–67]	60 [52–71]	0.565
Male, *n* (%)	21 (53.9%)	21 (53.9%)	1.000
Body mass index (kg/m^2^)	23.9 [21.5–26.5]	23.6 [21.4–24.8]	0.366
ASA-PS class I/II/III, *n* (%)	10 (25.6%)/ 16 (41.0%)/ 13 (33.3%)	9 (23.1%)/ 20 (51.3%)/ 10 (25.6%)	0.641
Smoking, non/ex/current, *n* (%)	32 (82.1%)/ 0 (0.0%)/ 7 (17.9%)	31 (79.5%)/ 3 (7.7%)/ 5 (12.8%)	0.187
Diabetes, *n* (%)	12 (30.77%)	12 (30.77%)	1.000
Hypertension, *n* (%)	17 (43.59%)	17 (43.59%)	1.000
Asthma or COPD, *n* (%)	4 (10.3%)	5 (12.8%)	1.000
Obstructive sleep apnea, *n* (%)	6 (15.4%)	7 (17.9%)	1.000
Hematocrit (%)	39.2 [36.3–43.0]	38.9 [35.4–43.2]	0.791
Platelet (× 10^3^/μL)	207 [184–260]	215 [181–251]	0.803
Creatinine (mg/dL)	0.89 [0.66–2.32]	0.79 [0.62–1.04]	0.389
Baseline SpO_2_ (%)	98 [97–99]	98 [98–99]	0.652
Type of regional anesthesia			0.735
Brachial plexus block, *n* (%)	35 (89.7%)	33 (84.6%)	
Spinal anesthesia, *n* (%)	4 (10.3%)	6 (15.4%)	
Sedative dose			
Induction dose of DEX (μg)	60 [50–70]	0	
Total dose of DEX (μg)	85 [67–107]	0	
Induction dose of RMMZ (mg)	0	2.5 [2.4–4.0]	
Total dose of RMMZ (mg)	0	13.5 [9.3–21.2]	
Fluid administration (ml)	100 [50–175]	100 [50–225]	0.192
Surgery time (min)	55 [35–80]	60 [30–88]	0.538

Continuous data are presented as medians [interquartile ranges], and categorical data are presented as n (%). DEX, dexmedetomidine; RMMZ, remimazolam; ASA-PS, American Society of Anesthesiologists physical status; COPD, chronic obstructive pulmonary disease; SpO_2_, peripheral oxygen saturation.

### 3.2. Primary outcome

**FIGURE 2:**
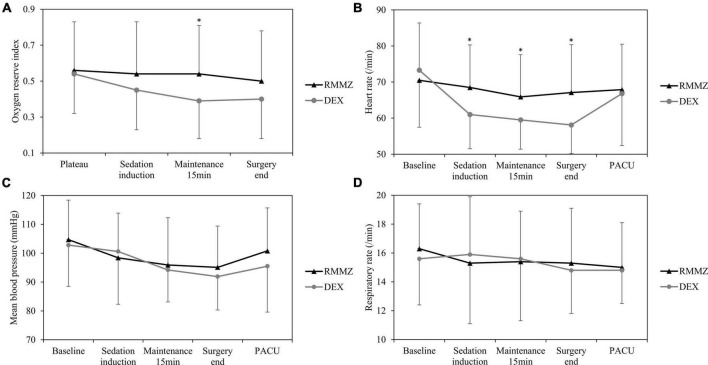
Perioperative **(A)** oxygen reserve index (ORi), **(B)** heart rate, **(C)** mean blood pressure, and **(D)** respiratory rate. Plateau means the maximum value of ORi in the preoxygenation stage. Sedation induction means after induction of sedation. Maintenance 15 min means 15 min after maintenance of sedation. *Significant differences. RMMZ, remimazolam; DEX, dexmedetomidine; PACU, post-anesthesia care unit.

The ORi was significantly higher in the RMMZ group at 15 min after sedation maintenance. After the induction of sedation and at the end of surgery, the ORi tended to be higher in the RMMZ group; however, the difference was not statistically significant (Figure 2A; Supplementary Table 3).

### 3.3. Secondary outcome

No significant difference was observed between the two groups in respiratory depression during sedation (Table 2). Intraoperative HR was significantly higher in the RMMZ group after induction of sedation, 15 min after sedation maintenance, and at the end of surgery. However, HR was not significantly different in the PACU (Figure 2B). There was no significant difference in mean BP and RR between the two groups (Figures 2C, D; Supplementary Table 3). The incidences of intraoperative hypo- or hypertension were comparable between the groups. There were no significant differences between the groups in the PACU LOS, incidence of PONV, delirium, AKI, and postoperative hospital LOS (Table 2).

**TABLE 2:** Perioperative outcomes of the study cohort.

	DEX (*n* = 39)	RMMZ (*n* = 39)	*p*-value
Respiratory depression, *n* (%)	6 (15.4%)	5 (12.8%)	1.000
Decreased ORi of > 50%, *n* (%)	17 (43.6%)	7 (17.9%)	0.027*
Intraoperative hypotension, *n* (%)	10 (25.6%)	10 (25.6%)	1.000
Intraoperative hypertension, *n* (%)	2 (5.1%)	4 (10.3%)	0.671
PACU LOS (min)	32 [31–45]	33 [31–37]	0.886
PONV, *n* (%)	4 (10.3%)	7 (17.9%)	0.515
Delirium, *n* (%)	1 (2.6%)	0 (0.0%)	1.000
Acute kidney injury, *n* (%)	1 (2.6%)	0 (0.0%)	1.000
Postoperative hospital LOS (day)	1 [1–1]	1 [1–2]	0.120

Continuous data are presented as medians [interquartile ranges], and categorical data are presented as *n* (%). *Statistically significant (*p* < 0.05). DEX, dexmedetomidine; RMMZ, remimazolam; ORi, oxygen reserve index; PACU, post-anesthesia care unit; LOS, length of stay; PONV, postoperative nausea and vomiting.

The number of patients with a decrease in the intraoperative ORi to < 50% was significantly higher in the DEX group (Table 2). Univariate logistic regression revealed that low baseline SpO_2_ and DEX were associated with a decrease in the intraoperative ORi to < 50%. In the multivariate logistic regression, DM, low baseline SpO_2_, and DEX were associated with a decrease in the intraoperative ORi to < 50% (Table 3). In the ROC curve analysis of intraoperative ORi to < 50%, the optimal cutoff value of the initial SpO_2_ was 97, with an area under the curve of 0.65 [0.51–0.79], sensitivity of 0.45, specificity of 0.82, positive predictive value of 0.52, and negative predictive value of 0.77.

**TABLE 3:** Univariate and multivariate logistic regression analyses of factors associated with a decrease in the intraoperative ORi to < 50%.

	Univariable		Multivariable	
	OR [95% CI]	*p*-value	OR [95% CI]	*p*-value
Female	0.98 [0.37–2.58]	0.970		
Age	1.00 [0.97–1.03]	0.787	0.98 [0.94–1.01]	0.225
**ASA-PS**				
I	Reference			
II	0.72 [0.21–2.55]	0.603		
III	1.39 [0.39–5.20]	0.612		
**Smoking status**				
None	Reference		Reference	
Ex-smoker	1.00 [0.04–11.02]	1.000	1.04 [0.03–17.70]	0.979
Current smoker	0.40 [0.06–1.69]	0.263	0.17 [0.02–1.23]	0.106
Body mass index	0.99 [0.86–1.15]	0.936		
Diabetes mellitus	2.67 [0.96–7.48]	0.058	5.59 [1.45–25.78]	0.017*
Asthma or COPD	1.14 [0.22–4.78]	0.859	0.93 [0.11–7.27]	0.941
Obstructive sleep apnea	1.51 [0.41–5.15]	0.512	1.34 [0.23–7.33]	0.734
Hematocrit	0.99 [0.95–1.01]	0.631		
Baseline SpO_2_	0.59 [0.38–0.87]	0.011*	0.58 [0.35–0.90]	0.022*
Plateau ORi	1.94 [0.27–14.53]	0.509		
DEX group	3.53 [1.30–10.48]	0.017*	4.58 [1.36–18.15]	0.019*
**Regional anesthesia**				
Spinal anesthesia	Reference		Reference	
Brachial plexus block	1.91 [0.43–13.38]	0.436	3.00 [0.40–45.05]	0.346

*Statistically significant (*p* < 0.05). ORi, oxygen reserve index; OR, odds ratio; CI, confidence interval; ASA-PS, American Society of Anesthesiologists physical status; COPD, chronic obstructive pulmonary disease; SpO_2_, peripheral oxygen saturation; DEX, dexmedetomidine; OS, orthopedic surgery; GS, general surgery.

## 4. Discussion

In many previous randomized controlled trials, RMMZ was reported to cause less respiratory depression than propofol (34–40). Tang et al. (41) reported that respiratory depression and hypoxia occurred less frequently with RMMZ than those with traditional sedative drugs. When administered as a sedative, DEX is also known to have insignificant depressive effects on respiration (42, 43). Kim et al. (44) reported that in spinal anesthesia, respiratory depression was more frequent in RMMZ than in DEX during intraoperative sedation (21.2 vs. 2.0%; *p* = 0.002); this result differs from the results of the current study. However, few studies have compared the respiratory effects of administering RMMZ and DEX as sedatives, and further studies are required to elucidate these effects in the future. The current study will be a valuable reference for further research. In this study, we confirmed respiratory depression using a relatively safe method of using the decrease in ORi; moreover, the ORi tended to be higher in the RMMZ group. However, the incidence of respiratory depression during sedation was comparable between the two groups. ORi is a relatively recently developed index, and further research is needed to determine whether a reduction in ORi is associated with respiratory depression and specifically to determine what percentage of reduction is clinically significant. Severe respiratory depression did not occur in either group in the current study, and both drugs were safely used for sedation.

Chae et al. (17) reported the optimal dose of RMMZ decreased with age based on the 95% effective doses during the induction of general anesthesia. In the present study, the dose of RMMZ administered until the induction of sedation reached an MOAA/S scale score of 3 also tended to decrease with age. Therefore, dose reduction should be considered when administering RMMZ to induce sedation in patients of advanced age.

In present study, DM was among the factors associated with a decrease in ORi to < 50%. Patients with DM may have an impaired response to hypoxia, which can be accompanied by foot ulcers, nephropathy, and retinopathy (45). When the participants in the present study were analyzed according to DM (non-DM: *n* = 54; DM: *n* = 24), there were no significant differences in baseline SpO_2_ (non-DM: 98 [98–99]; DM: 98 [97–99]; *p* = 0.448), plateau ORi in the preoxygenation phase (non-DM: 0.55 [0.40–0.78]; DM: 0.48 [0.33–0.65]; *p* = 0.291), and respiratory depression (non-DM: 9 (16.7%); DM: 2 (8.3%); *p* = 0.533) during sedation. Therefore, further research is required to determine whether DM is associated with ORi reduction owing to an impaired response to hypoxia, deteriorating oxygenation, or measurement issues due to peripheral blood flow impairment caused by DM.

Several studies have reported DEX-induced bradycardia (46, 47). Studies have also reported that there is less reduction in HR when RMMZ is administered than that with the administration of other anesthetic drugs (48, 49). Similar results were obtained in the present study. Therefore, RMMZ can be considered as a sedative for patients with concerns of bradycardia during surgery. Additionally, when using DEX, a 10 min loading procedure is required, and this time should be measured using a drug infusion device or alarm clock (50). Bradycardia is common during this procedure, and the anesthesiologist must closely observe patients’ vital signs (46, 47). RMMZ does not require a loading procedure; therefore, it can be administered more easily in clinical settings than DEX. For price comparison, based on this study, an average of 85 μg of DEX was administered during a 1 h surgery under sedation, and the cost for one vial of DEX is approximately $34.4 (United States dollars [USD]) in our institution. When using RMMZ, an average of 13.5 mg is administered, which costs approximately $17.6 USD per 20 mg vial. Costs may vary depending on the surgical and anesthetic situation and institution. Based on this study, RMMZ can reduce costs by approximately half compared with DEX. However, DEX is known to have an analgesic effect, whereas RMMZ is known to have a minimal analgesic effect (51). Based on these characteristics, further research should be conducted to evaluate the overall satisfaction with each drug for surgeons and patients.

## 5. Limitations

This study had several limitations. First, the authors arbitrarily set the 50% ORi reduction criterion. The decrease in ORi according to the degree of respiratory depression has not yet been reported, and it does not necessarily mean respiratory depression. For patients with a higher ORi, the information from this study can be used to reduce oxygen supplementation to decrease the degree of hyperoxia. Further studies are required to determine the extent to which the ORi is clinically significant. Second, double blinding could not be achieved because of the significant differences in drug infusion methods between DEX and RMMZ. To compensate for this limitation, the postoperative outcome assessor was blinded to the group assignment. Third, the types of surgery were not uniform. However, there was no significant difference in surgical site or type between the two groups. Several types of surgeries were included to generalize the study results to patients undergoing sedation. Fourth, as this study was a single center study with a relatively small sample size, our results cannot be generalized to other institutes and did not show significant differences in postoperative outcomes. We plan to perform a further large-scale multicenter study, and we expect to show significant differences in postoperative outcomes and intraoperative ORi at more time points. Finally, the degree of sedation could not be quantified objectively using the depth of anesthesia monitoring equipment, such as the bispectral index, since equipment that incurred additional costs was difficult to access. To compensate for this limitation, the degree of sedation was maintained at the same level in both groups, using the MOAA/S scale.

## 6. Conclusion

When RMMZ was used as a sedative, the ORi was higher at 15 min after sedation maintenance than when DEX was used. Factors associated with a decrease in the intraoperative ORi to < 50% were the use of DEX, low baseline SpO_2_, and DM. A baseline SpO_2_ of < 97% can predict a decrease in intraoperative ORi to < 50%. Intraoperative HR was higher with RMMZ than with DEX. RMMZ is recommended as a sedative for patients with low baseline SpO_2_ and concerns of possible bradycardia. Further large-scale multicenter randomized controlled trials should be conducted to evaluate the differences in respiratory aspects in sedation management between classical sedatives and RMMZ. Additionally, future studies on the degree of clinically significant reduction in ORi should be conducted.

## Data availability statement

The raw data supporting the conclusions of this article will be made available by the authors, without undue reservation.

## Ethics statement

The studies involving humans were approved by the Institutional Review Board of Kyung Hee University Hospital (KHUH 2023-02-036) on 17 March 2023. The studies were conducted in accordance with the local legislation and institutional requirements. The participants provided their written informed consent to participate in this study.

## Author contributions

SL: Conceptualization, Data curation, Formal analysis, Methodology, Project administration, Visualization, Writing – original draft. MSK: Conceptualization, Methodology, Writing – original draft. HK: Data curation, Software, Writing – original draft. J-HC: Conceptualization, Supervision, Writing – review and editing. MKK: Investigation, Supervision, Writing – review and editing. AY: Conceptualization, Data curation, Investigation, Project administration, Resources, Supervision, Writing – review and editing.
