# Supplementary material for: Comparison of oxygen reserve index according to the remimazolam or dexmedetomidine for intraoperative sedation under regional anesthesia—A single-blind randomized controlled trial
Source: Front Med (Lausanne). 2023 Nov 15;10:1288243. doi: 10.3389/fmed.2023.1288243 (PMC10684752; doi:10.3389/fmed.2023.1288243)
Supplement: Supplementary file 1 [file Table_1.DOCX]

|  | DEX (n = 39) | RMMZ (n = 39) | *p*-value |
| --- | --- | --- | --- |
| Type of surgery |  |  | 0.848 |
| OS upper extremity |  |  |  |
| ORIF, n (%) | 14 (35.9%) | 15 (38.4%) |  |
| Mass excision, n (%) | 8 (20.5%) | 9 (23.1%) |  |
| Carpal tunnel release, n (%) | 4 (10.2%) | 2 (5.1%) |  |
| GS upper extremity |  |  |  |
| Arteriovenous fistula, n (%) | 9 (23.1%) | 7 (18.0%) |  |
| OS lower extremity |  |  |  |
| ORIF, n (%) | 2 (5.1%) | 1 (2.6%) |  |
| Knee arthroplasty, n (%) | 1 (2.6%) | 2 (5.1%) |  |
| GS lower extremity |  |  |  |
| EVLT, n (%) | 1 (2.6%) | 3 (7.7%) |  |

**Supplementary Table 1.** Detailed types of surgery.

Categorical data are presented as n (%). OS, orthopedic surgery; ORIF, open reduction and internal fixation; GS, general surgery; EVLT, endo venous laser therapy.
