# Supplementary material for: Comparison of oxygen reserve index according to the remimazolam or dexmedetomidine for intraoperative sedation under regional anesthesia—A single-blind randomized controlled trial
Source: Front Med (Lausanne). 2023 Nov 15;10:1288243. doi: 10.3389/fmed.2023.1288243 (PMC10684752; doi:10.3389/fmed.2023.1288243)
Supplement: Supplementary file 2 [file Table_2.DOCX]

|  | DEX (n = 39) | RMMZ (n = 39) | *p*-value |
| --- | --- | --- | --- |
| Baseline | 5 [5–5] | 5 [5–5] | 1.000 |
| After sedation induction | 3 [3–4] | 3 [3–4] | 0.652 |
| 15 min after maintenance | 3 [3–3] | 3 [3–4] | 0.475 |
| End of surgery | 3 [3–4] | 3 [3–4] | 0.355 |
| Post anesthesia care unit | 4 [4–5] | 4 [4–5] | 0.788 |

**Supplementary Table 2.** Intraoperative Modified Observer's Assessment of Alertness and Sedation scale.

Data are presented as the median [interquartile ranges]. DEX, dexmedetomidine; RMMZ, remimazolam.
