# Supplementary material for: Comparison of oxygen reserve index according to the remimazolam or dexmedetomidine for intraoperative sedation under regional anesthesia—A single-blind randomized controlled trial
Source: Front Med (Lausanne). 2023 Nov 15;10:1288243. doi: 10.3389/fmed.2023.1288243 (PMC10684752; doi:10.3389/fmed.2023.1288243)
Supplement: Supplementary file 3 [file Table_3.docx]

|  | DEX (n = 39) | RMMZ (n = 39) | *p*-value |
| --- | --- | --- | --- |
| Oxygen reserve index |  |  |  |
| Plateau | 0.54 ± 0.22 | 0.56 ± 0.27 | 0.658 |
| After sedation induction | 0.45 ± 0.22 | 0.54 ± 0.29 | 0.153 |
| 15 min after maintenance | 0.39 ± 0.21 | 0.54 ± 0.27 | 0.005* |
| End of surgery | 0.40 ± 0.22 | 0.50 ± 0.28 | 0.075 |
| Heart rate |  |  |  |
| Baseline (/min) | 73.3 ± 13.1 | 70.5 ± 13.0 | 0.338 |
| After sedation induction (/min) | 61.0 ± 9.5 | 68.5 ± 11.8 | 0.003* |
| 15 min after maintenance (/min) | 59.5 ± 8.1 | 65.9 ± 11.7 | 0.006* |
| End of surgery (/min) | 58.1 ± 7.9 | 67.1 ± 13.3 | 0.001* |
| Post anesthesia care unit (/min) | 66.8 ± 14.4 | 67.9 ± 12.6 | 0.714 |
| Mean blood pressure |  |  |  |
| Baseline (mmHg) | 102.8 ± 14.3 | 104.7 ± 13.7 | 0.547 |
| After sedation induction (mmHg) | 100.6 ± 13.3 | 98.4 ± 16.1 | 0.521 |
| 15 min after maintenance (mmHg) | 94.2 ± 11.1 | 95.9 ± 16.4 | 0.573 |
| End of surgery (mmHg) | 91.9 ± 11.6 | 95.1 ± 14.3 | 0.289 |
| Post anesthesia care unit (mmHg) | 95.5 ± 15.9 | 100.8 ± 14.9 | 0.133 |
| Respiratory rate |  |  |  |
| Baseline (/min) | 15.6 ± 3.2 | 16.3 ± 3.1 | 0.369 |
| After sedation induction (/min) | 15.9 ± 4.0 | 15.3 ± 4.2 | 0.509 |
| 15 min after maintenance (/min) | 15.6 ± 3.3 | 15.4 ± 4.1 | 0.787 |
| End of surgery (/min) | 14.8 ± 3.0 | 15.3 ± 3.8 | 0.601 |
| Post anesthesia care unit (/min) | 14.8 ± 2.3 | 15.0 ± 3.1 | 0.744 |

**Supplementary Table 3.** Intraoperative oxygen reserve index, heart rate, and mean blood pressure.

Data are presented as the mean ± standard deviation. * Statistically significant. DEX, dexmedetomidine; RMMZ, remimazolam.
